# cAMP Signaling Prevents Podocyte Apoptosis via Activation of Protein Kinase A and Mitochondrial Fusion

**DOI:** 10.1371/journal.pone.0092003

**Published:** 2014-03-18

**Authors:** Xiaoying Li, Hua Tao, Kewei Xie, Zhaohui Ni, Yucheng Yan, Kai Wei, Peter Y. Chuang, John Cijiang He, Leyi Gu

**Affiliations:** 1 Renal Division and Molecular Cell Lab for Kidney Disease, Renji Hospital, School of Medicine, Shanghai Jiaotong University, Shanghai, China; 2 Division of Nephrology, Icahn School of Medicine at Mount Sinai, New York, New York, United States of America; 3 Renal Section, James J Peter Veterans Affairs Medical Center, Bronx, New York, United States of America; Roswell Park Cancer Institute, United States of America

## Abstract

Our previous *in vitro* studies suggested that cyclic AMP (cAMP) signaling prevents adriamycin (ADR) and puromycin aminonucleoside (PAN)-induced apoptosis in podocytes. As cAMP is an important second messenger and plays a key role in cell proliferation, differentiation and cytoskeleton formation via protein kinase A (PKA) or exchange protein directly activated by cAMP (Epac) pathways, we sought to determine the role of PKA or Epac signaling in cAMP-mediated protection of podocytes. In the ADR nephrosis model, we found that forskolin, a selective activator of adenylate cyclase, attenuated albuminuria and improved the expression of podocyte marker WT-1. When podocytes were treated with pCPT-cAMP (a selective cAMP/PKA activator), PKA activation was increased in a time-dependent manner and prevented PAN-induced podocyte loss and caspase 3 activation, as well as a reduction in mitochondrial membrane potential. We found that PAN and ADR resulted in a decrease in *Mfn1* expression and mitochondrial fission in podocytes. pCPT-cAMP restored *Mfn1* expression in puromycin or ADR-treated podocytes and induced *Drp1* phosphorylation, as well as mitochondrial fusion. Treating podocytes with arachidonic acid resulted in mitochondrial fission, podocyte loss and cleaved caspase 3 production. Arachidonic acid abolished the protective effects of pCPT-cAMP on PAN-treated podocytes. Mdivi, a mitochondrial division inhibitor, prevented PAN-induced cleaved caspase 3 production in podocytes. We conclude that activation of cAMP alleviated murine podocyte caused by ADR. PKA signaling resulted in mitochondrial fusion in podocytes, which at least partially mediated the effects of cAMP.

## Introduction

Most glomerular diseases share the common features of proteinuria and effacement of podocyte foot processes [1]. Recent studies have shown that podocyte injury is involved in the development of podocytopenia and glomerulosclerosis [2]. The first event initiating the process of glomerulosclerosis is podocyte loss [3], [4]. The remnant podocytes fail to cover the naked GBM, resulting in the formation of tufts which adhere to each other or to the Bowman's capsule. Lack of proliferation and increased loss of podocytes contribute to a reduction in podocyte number [5]. Although kidney progenitor cells, glomerular parietal epithelial cells or stem cells may differentiate into podocytes [6]–[8], a large amount of evidence suggests that podocyte loss including detachment or apoptosis leads to reduced podocyte number (podocytopenia) in the disease state [9]–[11]. In our previous studies, we found that the TUNEL-positive podocyte number was inversely associated with the WT-1 positive cell number in the adriamycin (ADR) mouse model, indicating that apoptosis contributes, at least partially, to podocyte loss [12].

There are several signaling pathway involved in the apoptosis of podocytes, such as activation of the renin-angiotensin system, ROS production, and TGF-β signaling [13]–[16]. In addition to PI3K/AKT signaling [17], [18], we found that an increase in intracellular cyclic AMP (cAMP) level also prevented PAN-induced apoptosis of cultured podocytes [12]. cAMP is known to activate either protein kinase A (PKA) or cAMP-activated guanine nucleotide exchange factors for Ras-like GTPases (Epac) [19]. Little is known about c-AMP-mediated signaling pathways in podocyte function, although it has been shown that cAMP signaling mediates cell differentiation, actin assembly and matrix production in podocytes.

Recently, Zhu et al showed that mitochondrial dysfunction plays an important role in podocyte damage [20]. Cytochrome *c* and other pro-apoptosis factors released from the intermembrane space of injured mitochondria triggered the initiation of the mitochondrial pathway of apoptosis [21], [22]. The balance between morphologic changes in mitochondria, so-called fission and fusion, is essential for maintaining mitochondrial function [23]. However, mitochondria dynamics have not been previously investigated in podocytes.

The present study was designed to test whether cAMP signaling alleviates doxorubicin-induced nephrosis *in vivo* and to determine the role of PKA, Epac and morphologic changes in mitochondria in mediating the protective effects of cAMP against PAN-induced podocyte apoptosis.

## Materials and Methods

### Reagents

pCPT-cAMP, 8-pCPT-2-O-Me-cAMP, H89, puromycin aminonucleoside, mdivi-1 and arachidonic acid were purchased from Sigma Chemical (St Louis, MO, USA). Forskolin was purchased from Tocris (Bristol, UK). ADR was purchased from Enzo Life Sciences (Lausen, Switzerland).

### Animal experiments

All animal procedures were approved by Renji Hospital, Shanghai Jiaotong University School of Medicine. Male BalB/C mice (Shanghai Slac Laboratory Animal, China) aged 8–10 weeks were divided into the control, ADR model, and forskolin-treated ADR model groups. ADR nephropathy was induced by a single intravenous injection of 10 mg/kg ADR. Forskolin was administered by intraperitoneal injection at a dose of 5 mg/kg 1 h before ADR on day 1. Forskolin was then injected every other day. On day 14, urine was collected for 24 h using a metabolic cage (n = 6 per group). On day 15, the mice were killed under chloral hydrate anesthesia and the kidneys were removed for analyses.

### Transmission EM

Cortical kidney sections were fixed with 2.5% glutaraldehyde in 4°C phosphate-buffered saline overnight, post-fixed in 1.0% OsO4, and embedded in LR White resin (London Resin, Hampshire, UK). Ultrathin sections (70 nm) were stained with uranyl acetate and examined under an electron microscope (Philips CM120; Eindhoven, The Netherlands) at 75 kV. A pathologist analyzed the width of the sections containing at least 10 capillary loops per mouse, using the iTEM software (Olympus Soft Imaging Solutions GmbH, Munster, Germany) and a blinded method.

### Coomassie Blue stain for proteinuria

Albuminuria was measured using coomassie blue stain according to a previously described protocol [24].

### Urine creatinine detection

Urine creatinine concentration was determined using a creatinine assay kit (Cayman Chemical, Ann Arbor, MI, USA), according to the manufacturer's instructions. The creatinine concentration in urine samples was determined using a creatinine standard curve. The dynamic range of the kit was 0–15 mg/dl of creatinine. The absorbance at 500 nm was determined using a microplate reader.

### Podocyte culture

Conditionally immortalized mouse podocytes were a gift from Dr Peter Mundel and have been described previously. The majority of cells used in the present studies had an arborous shape and expressed synaptopodin. Differentiated podocytes were treated with various reagents. All experiments were repeated at least four times for each indicated condition. Podocytes between passages 9 and 20 were used in all experiments.

### Immunoblot analyses

Sodium dodecyl sulfate-polyacrylamide gel electrophoresis and immunoblotting analyses were carried out according to standard protocols and visualized using an Infrared Imaging System (Odyssey). We used rabbit anti-human caspase-3 antibody (1∶1000), rabbit anti-human cleaved caspase-3 antibody (1∶1000), rabbit anti-human Drp1 antibody (1∶1000), rabbit anti-human p (s637)-Drp1 antibody (1∶1000, Cell Signaling Technology, Danvers, MA, USA), rabbit anti-human Mfn1 antibody (1∶1000), rabbit anti-rat OPA1 antibody (1∶1000, Abcam, Cambridge, UK), and rabbit anti-human Fis1 antibody (1∶500, Enzo Life Sciences, Lausen, Switzerland).

### Immunofluorescence and Immunohistochemical staining

Cryosections 3-μm thick were prepared using a cryostat, and were fixed in 4% paraformaldehyde for 15 min at room temperature. After blocking, the cryosections were incubated with secondary primary antibodies followed by recognition with fluorescein Cy3/TRITC-labeled secondary antibodies (1∶400). Fluorescence images were recorded using a Leica TCS SP5II confocal microscope (Leica Microsystems, Buffalo Grave, IL, USA). The following primary antibodies were used: mouse anti-mouse synaptopodin (1∶20), rabbit anti-human Mfn1 antibody (1∶50), and rabbit anti-human p-CREB antibody (1∶50, Santa Cruz).

For immunohistochemistry, 3-μm-thick cryosections were prepared using a cryostat and fixed in 4% paraformaldehyde. After blocking, the cryosections were incubated with primary antibodies to WT-1 (1∶100, Santa Cruz). Sections were then incubated with horseradish peroxidase-labeled secondary antibodies (DAKO, Carpinteria, CA, USA).

### Mitochondrial staining

Mitochondrial morphology was detected using the CellLight Mitochondria-GFP Bacman2.0 according to the manufacturer's instructions. Briefly, we added the reagent directly to the cells which were incubated overnight for protein expression. The next day, the cells were visualized using a Leica TCS SP5II confocal microscope.

### Cell viability

Quantitative evaluation of cell viability was performed using a cell count kit-8 (Beyotime), according to the manufacturer's instructions. The absorbance at 450 nm was determined using a microplate reader.

### TUNEL staining

Cells that had entered advanced stages of apoptosis were detected by the TUNEL assay, performed with a commercial fluorometric TUNEL system kit (Promega, Madison, WI, USA), according to manufacturer's instructions.

### Determination of mitochondrial membrane potential

Mitochondrial membrane potential (△Ψm) was determined using the dual emission mitochondrial dye 5,5′,6,6′-tetrachloro-1,1′,3,3′-tetraethylbenzimidazolocarbocyanine iodide (JC-1, Beyotime). At low △Ψm, the monomeric form of JC-1, shows green fluorescence (emission∼529 nm), whereas within the mitochondrial matrix, at high △Ψm, JC-1 is able to form aggregates, and shows red fluorescence (emission∼590 nm). Mitochondrial membrane potential was quantified by flow cytometric (BD FACS-Calibur, Franklin Lakes, NJ, USA) determination of cells with green fluorescence.

### Determination of PKA activity

PKA activity was assayed using the Promega PepTag assay for nonradioactive detection of cAMP-dependent protein kinase (Promega, Madison, WI, USA). All PepTag assay reaction components were combined on ice, and PKA activity was assayed at 30°C for 20 min. The reaction was stopped by incubating the tubes at 95°C for 10 min. Samples were loaded onto 0.8% agarose gel. Electrophoresis was run at 110 V for 30 min and was imaged under UV light by Smartview 2.0 software.

### Statistical analyses

All results are expressed as mean ± s.d. Statistical analyses were performed using SPSS 11. Analysis of variance with Duncan's test and Dunnett's test were used to assess differences between multiple groups. P<0.05 was considered statistically significant.

## Results

### Forskolin alleviated ADR-induced proteinuria and podocyte injury

**Figure 1:**
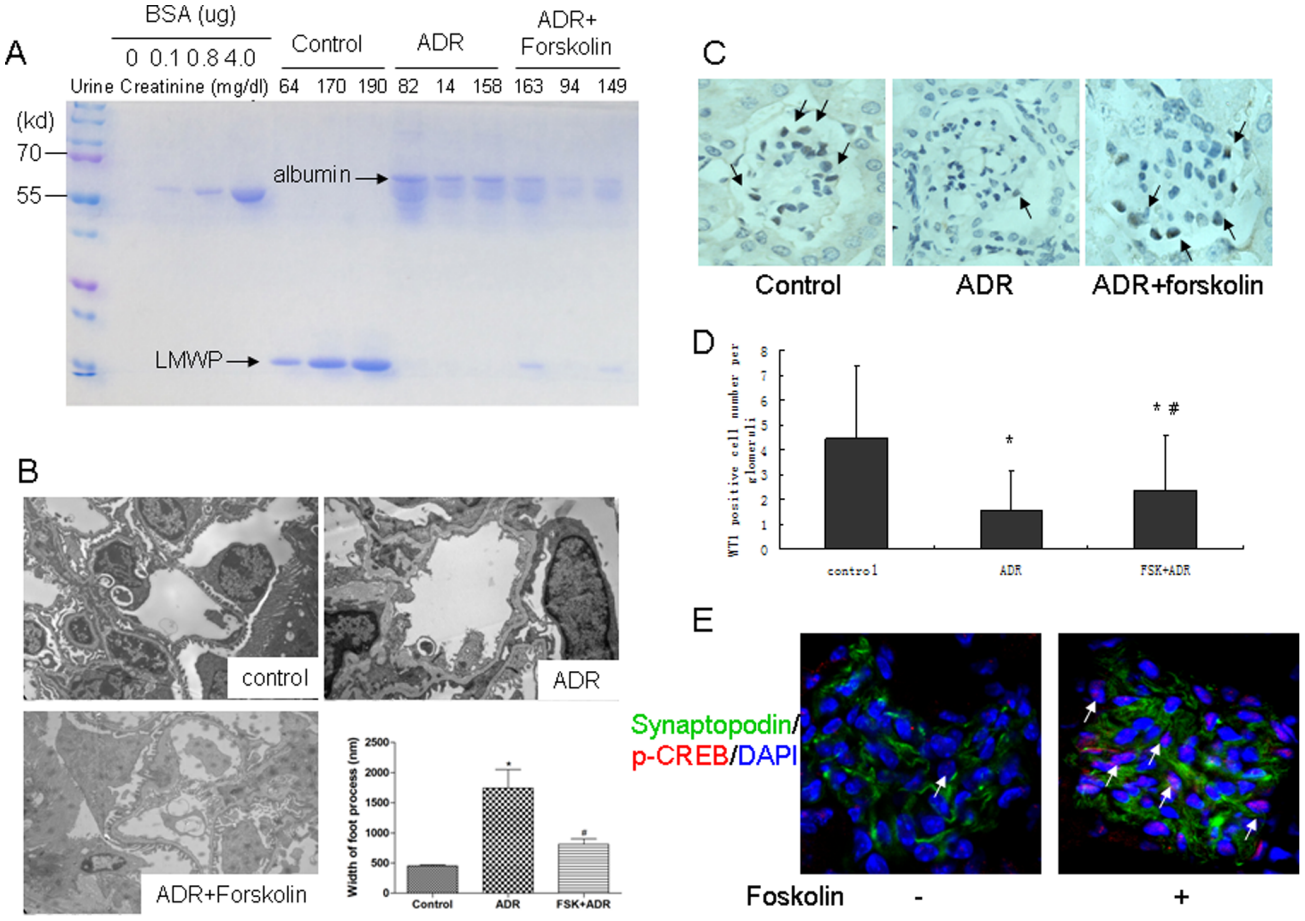
Forskolin attenuated ADR nephrosis. A: Coomassie blue staining of urine proteins resolved by gel electrophoresis. Creatinine concentration indicated to variable dilutions among urine samples. LMWP: low molecular weight protein; B: Electron microscopy of a capillary loop (×13500). C: Immunohistochemical staining was performed to detect WT-1 positive cells in glomeruli (×200). WT-1 positive cell numbers were counted by one renal pathologist using a blinded method. At least 50 glomeruli per kidney were calculated. Black arrow: WT-1 positive cells. D: A bar graph of the data expressed as average number per glomerulus. E: Immunofluorescence staining of kidney from mice treated with or without forskolin (×200). White arrow: p-CREB positive cells. *: P<0.05 compared with control group, #: P<0.05 compared with ADR group.

ADR significantly increased urinary albumin excretion, which was decreased in forskolin-pretreated mice (Figure 1A). Compared with the control group, the foot processes of podocytes in ADR-treated mice were effaced, retracted and widened. These ultrastructural changes and albuminuria were attenuated in the forskolin-treated group (Figure 1A and 1B). We calculated the average number of podocytes per glomerulus based on WT-1 staining. WT-1 positive cell numbers per glomerulus were 4.47±2.91, 1.58±1.63 and 2.37±2.24 (P<0.01) for the control, ADR and forskolin groups, respectively (Figure 1C and 1D), suggesting that forskolin partially prevented doxorubicin-induced podocyte loss. It is known that activation of PKA causes CREB phosphorylation. We found that phospho-CREB staining was increased in the forskolin-treated mice.

### cAMP protected against PAN-induced podocyte loss via PKA signaling

**Figure 2:**
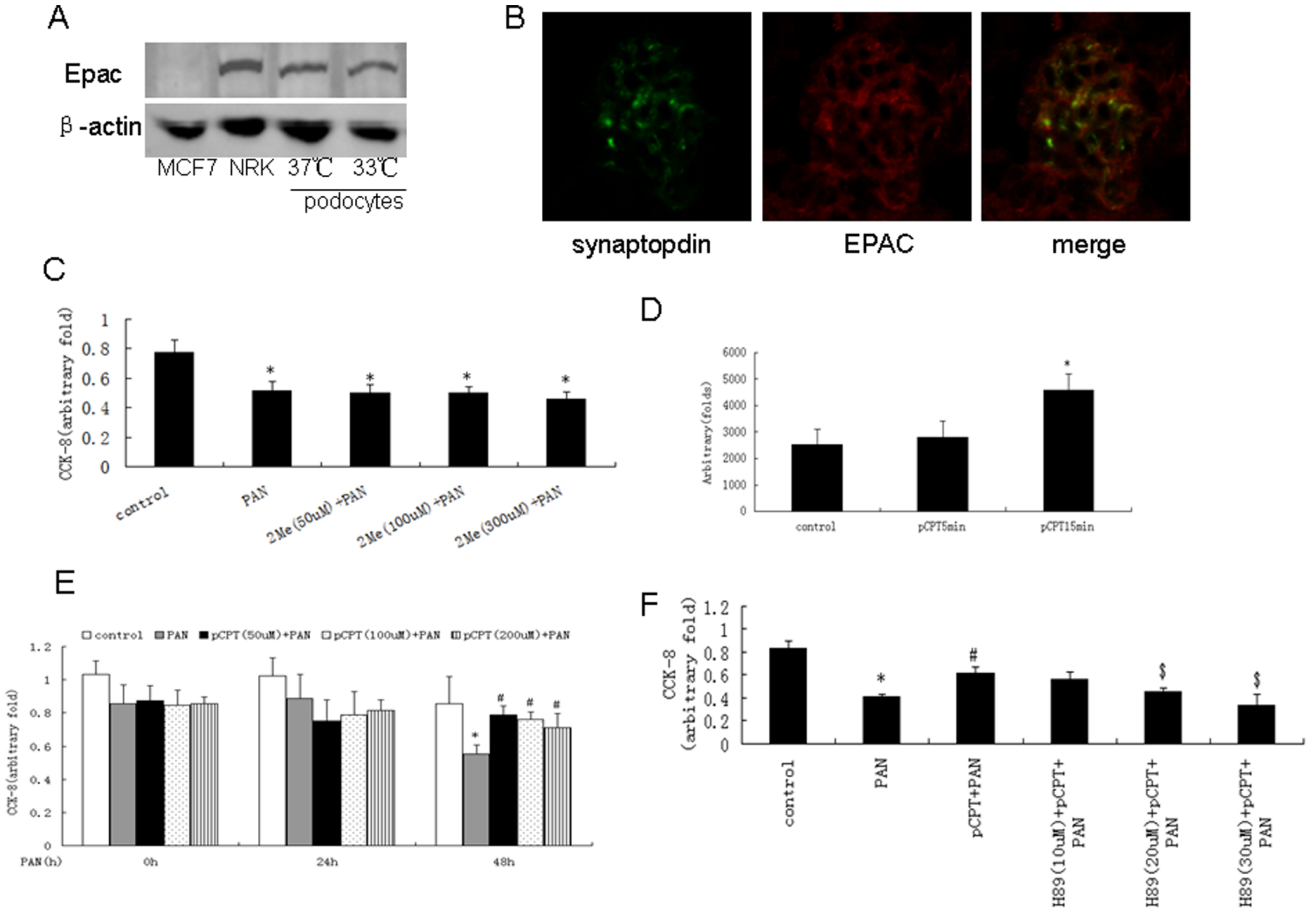
PKA-mediated cAMP protection against podocyte injury. A: Western blot of MCF7 cells, NRK cells, differentiated and undifferentiated podocytes. B: Immunofluorescence staining of kidney from control mice (×200). C: CCK-8 test was performed using podocytes treated with PAN in the presence or absence of 2Me-cAMP. Data were obtained from five independent studies. D: PKA activity was detected using podocytes treated with pCPT-cAMP for the time indicated. Data were obtained from five independent studies. E: CCK-8 test was performed using podocytes treated with PAN in the presence or absence of pCPT-cAMP. Data were obtained from five independent studies. F: CCK-8 results from podocytes treated with PAN in the presence or absence of pCPT-cAMP and H89. Data were obtained from five independent studies. *: P<0.05 compared with control group, #: P<0.05 compared with PAN group, $: P<0.05 compared with pCPT+PAN group. 2Me: 2Me-cAMP, pCPT: pCPT-cAMP.

**Figure 3:**
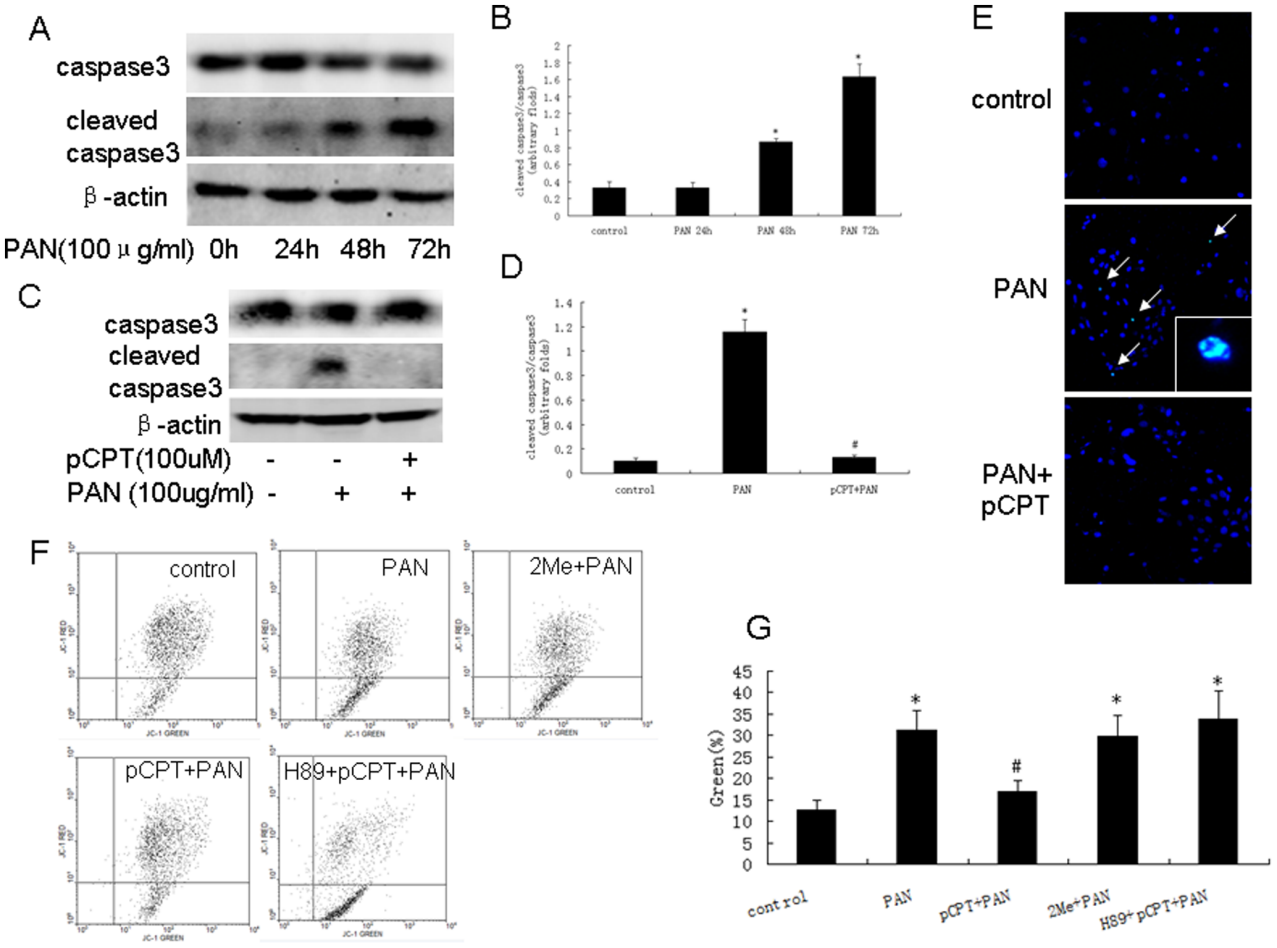
PKA signaling prevented PAN-induced podocyte apoptosis. A and B: Western blot of podocytes treated with PAN for the indicated time. Bar graph of data obtained from five independent experiments. C and D: Western blot for podocytes treated with PAN in the presence or absence of pCPT-cAMP. Bar graph of data obtained from five independent experiments. E: TUNEL staining results (×100). White arrows indicate the TUNEL positive cells. F and G: JC-1 staining and bar graph of data obtained from four to five independent experiments. *: P<0.05 compared with control group, #: P<0.05 compared with PAN group. 2Me: 2Me-cAMP, pCPT: pCPT-cAMP.

As shown in Figure 2A and 2B, Epac was expressed in both differentiated and undifferentiated podocytes. However, 2Me-cAMP (cAMP/Epac mimic) failed to prevent PAN-induced cell loss (Figure 2C). As shown in Figure 2D and 2E, pCPT-cAMP activated PKA in a time-dependent manner and prevented against PAN-induced podocyte loss. pCPT-cAMP did not prevent podocyte loss when podocytes were pretreated with H89 (PKA inhibitor) (Figure 2F). PAN treatment resulted in an increase in cleaved caspase 3, which was blocked by pCPT-cAMP pretreatment (Figure 3A to 3D). Similar results were observed by TUNEL staining (Figure 3E). We used JC-1 staining to show that pretreatment with pCPT-cAMP, but not 2Me-cAMP, prevented the decrease in mitochondrial membrane potential in PAN-treated podocytes (12.67±2.15%, 31.35±4.60%, 16.96±2.51% and 29.85±4.87% of podocytes exhibited green fluorescence in the control, PAN, PAN+pCPT-cAMP and PAN+2Me-cAMP groups, respectively) (Figure 3F). pCPT-cAMP failed to prevent the decrease in mitochondrial membrane potential in podocytes pre-treated with H89 (Figure 3G).

### pCPT-cAMP pretreatment prevented mitochondrial division in PAN-treated podocytes

**Figure 4:**
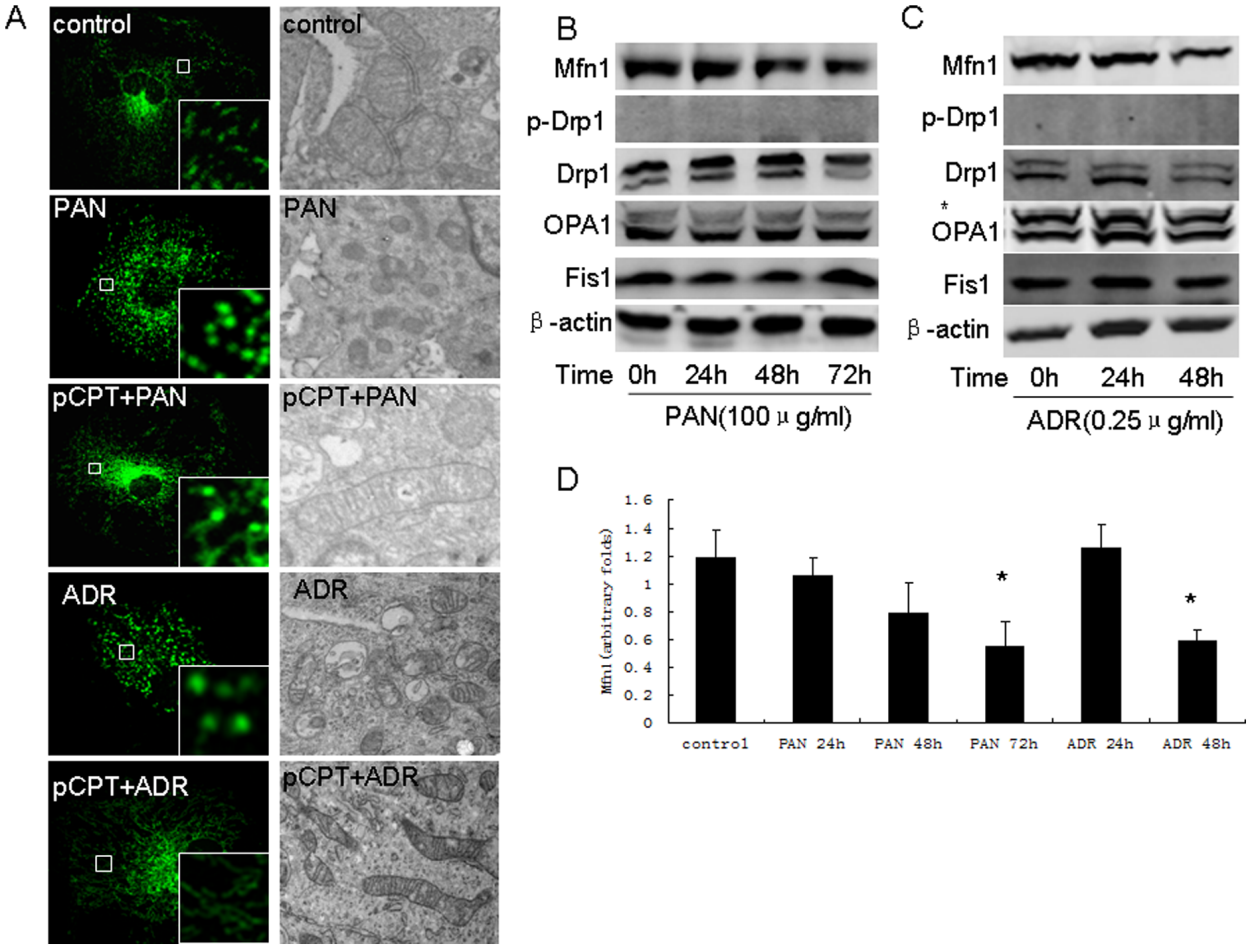
Mitochondria fission in podocytes was induced by PAN or ADR treatment. A: Mitochondria staining (×200) and electron microscopy (×17500) of podocytes treated with PAN in the presence or absence of pCPT-cAMP. B: Western blot of podocytes treated with PAN for the indicated time. C: Bar graph of data from five independent experiments. D: Western blot of podocytes treated with PAN for the indicated time. *: P<0.05 compared with control group. pCPT: pCPT-cAMP.

**Figure 5:**
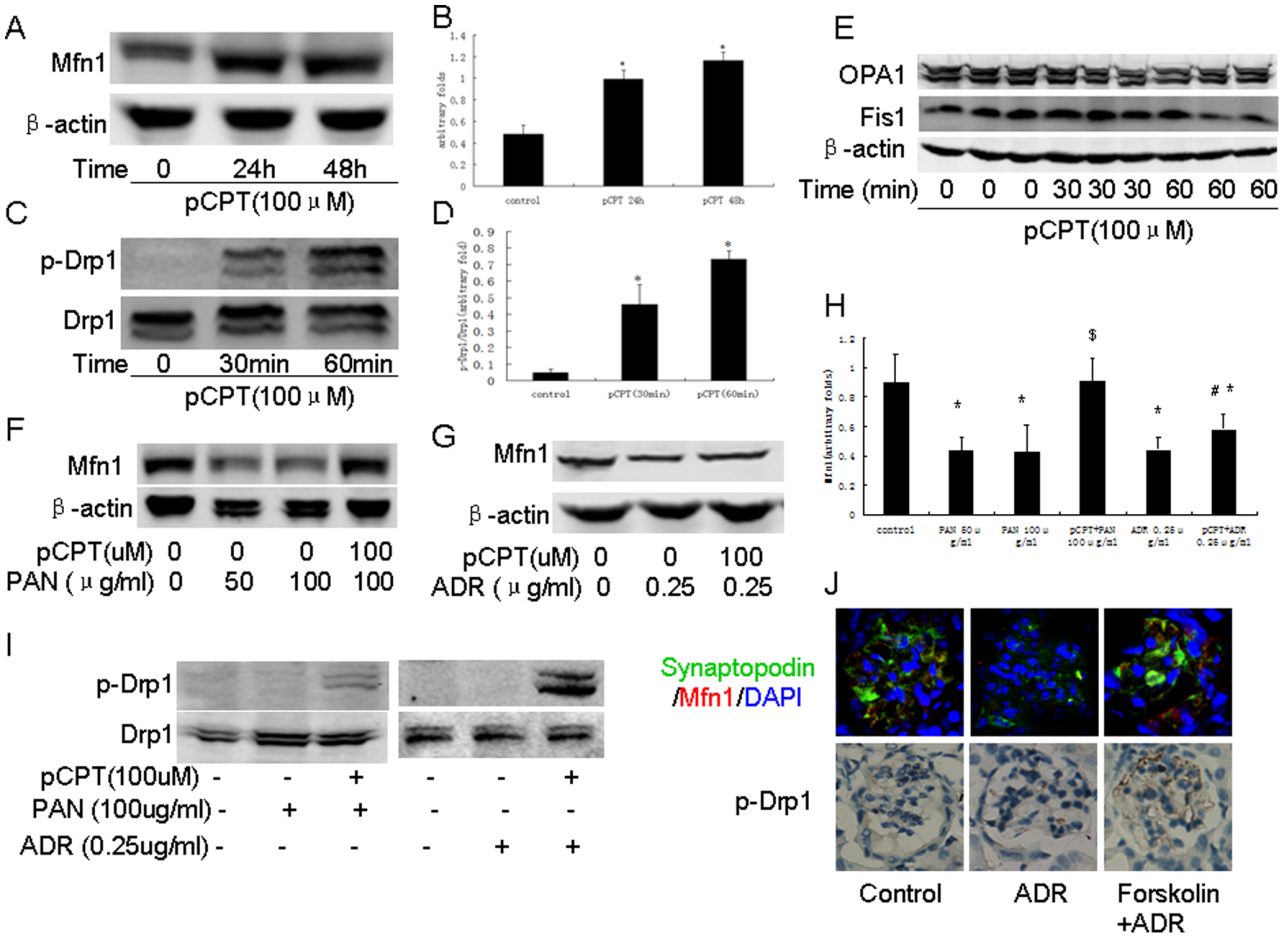
PKA signaling promoted mitochondria fusion in podocytes. A and B: Western blot of podocytes treated with pCPT-cAMP for the indicated time. Bar graph of data obtained from five independent experiments. C and D: Western blot results from podocytes treated with pCPT-cAMP for 30 and 60 minutes. Bar graph of data obtained from at least five independent experiments. E: Western blot performed for podocytes treated with pCPT-cAMP for the indicated time. F and G: Western blot performed for podocytes treated with PAN or ADR in the presence or absence of pCPT-cAMP. H: Bar graph of data from five independent experiments. I: Western blot performed for podocytes treated with PAN or ADR in the presence or absence of pCPT-cAMP. J: Immunofluorescence and immunohistochemical staining of kidney from the control, ADR and forskolin+ADR groups (×200). *: P<0.05 compared with control group, #: P<0.05 compared with PAN group. pCPT: pCPT-cAMP.

Mitochondria dynamics is regulated by Drp1, Fis1 (both trigger mitochondria fission), mitofusion and opa1 (both control mitochondria fusion). We estimated these protein expressions. As shown in Figure 4A, PAN or ADR treatment resulted in mitochondrial division. The mitochondria appeared longer in pCPT-cAMP pretreated podocytes. We found that mitofusin (Mfn) 1 protein was inhibited by PAN or ADR in a time-dependent manner. There was no change in the phosphorylation of Drp1 or expression of OPA1 and Fis1 (Figure 4B, 4C and 4D). As shown in Figure 5J, we also found that Mfn1 expression was decreased in ADR-treated mice, but not in foskolin+ADR-treated mice. Phosphorylated Drp1 was increased in foskolin+ADR-treated mice, however, no change was observed in ADR-treated mice.

pCPT-cAMP induced rapid phosphorylation of Drp1 in both control podocytes (Figure 5C and 5D) and PAN or ADR-treated podocytes (Figure 5I). pCPT-cAMP treatment also up-regulated Mfn1 expression in a time-dependent manner (Figure 5A and 5B) and restored Mfn1 expression in PAN or ADR-treated podocytes (Figure 5F, 5G and 5H).

### Induction of mitochondrial fission results in podocyte injury

**Figure 6:**
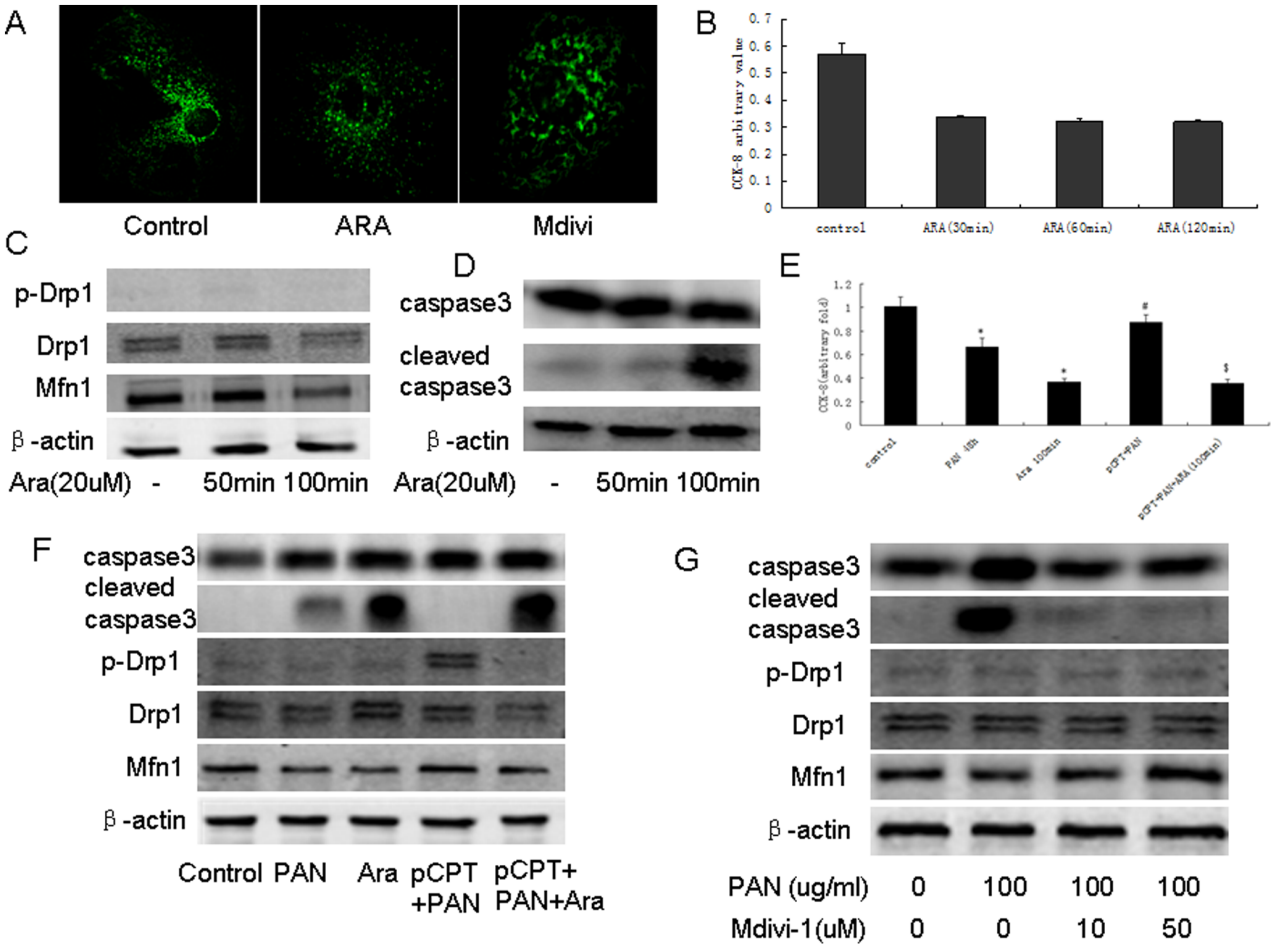
Mitochondria fusion/fission was involved in podocyte apoptosis. A: Mitochondria staining of podocytes treated with ARA or Mdivi. B: CCK-8 results from podocytes treated with ARA for the indicated time. Data were obtained from five independent experiments. C: Western blot of podocytes treated with ARA for the indicated time. D: CCKD-8 was performed using podocytes incubated with the indicated reagents. Data from five independent experiments are shown. E: Western blot of podocytes treated with the indicated reagents. F: Western blot of podocytes treated with PAN in the presence or absence of Mdivi-1. *: P<0.05 compared with control group, #: P<0.05 compared with PAN group, $: P<0.05 compared with pCPT+PAN group. pCPT: pCPT-cAMP.

We used arachidonic acid (ARA) or Mdivi-1 to induce mitochondrial fission or fusion in podocytes (Figure 6A) and found that treatment of podocytes with ARA resulted in cell loss (Figure 6B) and increased cleavage of caspase 3 (Figure 6C). As shown in Figure 6D and 6E, the protective effects of pCPT/cAMP were lost when cells were pretreated with ARA. PAN-induced expression of cleaved caspase 3 was partially inhibited in podocytes pretreated with Mdivi-1 (Figure 6F).

## Discussion

Podocytes are terminally differentiated cells and play an important role in maintaining glomerular permeability [2]. Podocyte injury or loss has been found in various glomerular diseases [1]–[3]. It is well known that vasoactive hormones, such as angiotensin II regulate cell function via ligand-receptor binding [25]. Podocytes express a variety of G-protein-coupled receptors, including metabotropic glutamate receptor 1, adrenergic β2 and Dopamine D1, which mediate an increase in cAMP upon stimulation [12], [26], [27]. Several lines of evidence indicate that the cAMP pathway affects podocyte biology. cAMP mediates the anti-proliferative and pro-differentiation effects of retinoic acid [28] and an increase in intracellular cAMP resulted in protection from puromycin-induced podocyte loss [12]. Gao et al. showed that cAMP promotes the assembly of cell-cell contacts between podocytes [29]. In recent studies, Keravis et al [30] demonstrated that phosphodiesterase type 4 (PDE4) inhibitors, which are known to increase cAMP level by preventing degradation, ameliorate the progression of lupus nephritis in mouse models. These data suggest that cAMP signaling has an overall glomerulo-protective effect [31]. In the present study, we showed that the direct activation of adenylate cyclase with forskolin can partially attenuate doxorubicin-induced albuminuria and podocyte loss. We expect that a more pronounced effect would be observed if we combined forskolin with a PDE4 inhibitor, which would induce a higher and more sustained level of cAMP in podocytes as this was previously shown with retinoic acid and a PDE4 inhibitor [32].

We demonstrated that podocyte express both PKA and Epac, the downstream molecule of cAMP. Previous studies have suggested that activation of the PKA pathway protects podocytes from injury. All-trans retinoic acid restores differentiation markers in HIV-infected podocytes via activation of the cAMP/PKA signaling pathway and the expression of nephrin in podocytes is regulated by cAMP/PKA [27], [33]. Oba S et al. found that activation of PKA prevents PAN-induced ROS generation in podocytes [34]. However, there are no published studies on the role of Epac in podocyte biology and pathology, although increasing evidence suggests that Epac plays an important role in other cell types [35]. Li Y et al. showed that Epac is mainly expressed in tubular epithelial cells and weakly expressed in podocytes of human glomeruli, but it is absent in rat glomeruli [36]. Activation of Epac signaling protects proximal tubular epithelial cells from cisplatin-induced apoptosis and ischemia-induced kidney failure [37], [38]. Our data showed that Epac is expressed in mouse glomeruli and tubuli, but we were unable to identify the role of Epac in PAN-induced podocyte loss using a cAMP/Epac agonist. A small GTPase, Rap, is the downstream molecule of Epac [35], and we suspect that Epac signaling may be involved in regulation of the cytoskeleton instead of cell cycle/apoptosis in podocytes.

In the present research, we focused on the role of PKA and Epac. However, other signaling molecules may crosstalk with the cAMP pathway. Faour WH et al. found that neither PKA nor Epac mediated forskolin/IBMX-induced p38 activation and COX-2 expression in podocytes [39]. cAMP treatment also induced activation of TRPC6 independent of PKA or Epac in HEK293 cells [40].

We focused on mitochondrial dysfunction as mitochondria have a central role in the initiation of apoptosis. The release of cytochrome *c* from damaged mitochondria may activate caspase 9 and downstream caspases. We found that more cells developed mitochondrial fission in PAN-treated podocytes than in control cells. Usually, mitochondrial fission indicates that cells need more ATP or need to remove more damaged mitochondria. We did not find Drp1 upregulation in PAN-treated podocytes, although mitochondria fission is triggered by Drp1. It is interesting that mitofusin-1, which controls mitochondrial fusion was down-regulated after PAN treatment, suggesting that PAN-induced mitochondrial fission was a passive process in podocytes. Mitochondrial fusion allows healthy mitochondria to provide intact mitochondrial components, including DNA and proteins, to damaged mitochondria to maintain normal mitochondrial function, thus preventing mitochondrial outer membrane rupture and the release of apoptogenic factors [41].

It is well known that cAMP-dependent protein kinase (PKA) phosphorylates Ser637 of Drp1, and then phosphorylated Drp1 is released from mitochondria to the cytosol, leading to mitochondria fusion and suppression of cell apoptosis [41]. In the present study, forskolin or pCPT-cAMP induced Ser637 phosphorylation of Drp1 expression in both *in vivo* and *in vitro* studies. In addition to Drp1 phosphorylation, mitofusin-1 expression was also upregulated by forskolin or pCPT-cAMP treatment. As mitofusin is the target gene of peroxisome proliferator activated receptor γ coactivator-1α (PGC-1α) [42], [43] and PGC-1α expression is regulated by PKA, we think that PKA/PGC-1α signaling mediated forskolin-induced mitofusin-1 up-regulation. In fact, Cowell et al. found that mitofusin-1 gene expression was up-regulated 2.2-fold and 2.3-fold in forskolin-treated and PGC-1α overexpressed Schwann cells, respectively [42]. These data suggest that cAMP/PKA signaling not only promotes mitochondrial fusion by increasing the phosphorylation of Drp1, but also prevents PAN-induced mitochondrial fission by recovering mitofusin expression.

### Conclusion

We conclude that PKA, but not Epac, signaling mediates the protective effects of cAMP in podocytes, which at least partially via induction of mitochondrial fusion. These results provide insight into the mechanism of cAMP in podocytes and help us to identify potential new targets for the treatment of glomerular disease.

## References

[1] Johnstone DB, Holzman LB (2006) Clinical impact of research on the podocyte slit diaphragm. Nat Clin Pract Nephrol 2: 271–282. 16932440 10.1038/ncpneph0180

[2] Pavenstadt H, Kriz W, Kretzler M (2003) Cell biology of the glomerular podocyte. Physiol Rev 83: 253–307. 12506131 10.1152/physrev.00020.2002

[3] Kriz W, Gretz N, Lemley KV (1998) Progression of glomerular diseases: is the podocyte the culprit? Kidney Int 54: 687–697. 9734594 10.1046/j.1523-1755.1998.00044.x

[4] Kriz W, Hosser H, Hahnel B, Gretz N, Provoost AP (1998) From segmental glomerulosclerosis to total nephron degeneration and interstitial fibrosis: a histopathological study in rat models and human glomerulopathies. Nephrol Dial Transplant 13: 2781–2798. 9829480 10.1093/ndt/13.11.2781

[5] Shankland SJ (2006) The podocyte's response to injury: Role in proteinuria and glomerulosclerosis. Kidney Int 69: 2131–2147. 16688120 10.1038/sj.ki.5000410

[6] Ronconi E, Sagrinati C, Angelotti ML, Lazzeri E, Mazzinghi B, et al (2009) Regeneration of glomerular podocytes by human renal progenitors. J Am Soc Nephrol 20: 322–332. 19092120 10.1681/ASN.2008070709 PMC2637058

[7] Appel D, Kershaw DB, Smeets B, Yuan G, Fuss A, et al (2009) Recruitment of podocytes from glomerular parietal epithelial cells. J Am Soc Nephrol 20: 333–343. 19092119 10.1681/ASN.2008070795 PMC2637040

[8] Prodromidi EI, Poulsom R, Jeffery R, Roufosse CA, Pollard PJ, et al (2006) Bone marrow-derived cells contribute to podocyte regeneration and amelioration of renal disease in a mouse model of Alport syndrome. Stem Cells 24: 2448–2455. 16873763 10.1634/stemcells.2006-0201

[9] Petermann A, Krofft R, Blonski M, Hiromura K, Vaughn M, et al (2003) Podocytes that detach in experimental membranous nephropathy are viable. Kidney Int 64: 1222–1231. 12969140 10.1046/j.1523-1755.2003.00217.x

[10] Yu D, Petermann A, Kunter U, Rong S, Shankland SJ, et al (2005) Urinary podocyte loss is a more specific marker of ongoing glomerular damage than proteinuria. J Am Soc Nephrol 16: 1733–1741. 15829708 10.1681/ASN.2005020159

[11] Ding G, Reddy K, Kapasi AA, Franki N, Gibbons N, et al (2002) Angiotensin II induces apoptosis in rat glomerular epithelial cells. Am J Renal Physiol 283: F173–F180. 10.1152/ajprenal.00240.2001 12060599

[12] Gu L, Liang X, Wang L. Yan Y, Ni Z, et al (2012) Functional metabotropic glutamate receptors 1 and 5 are expressed in murine podocytes. Kidney Int 81: 458–468. 22166849 10.1038/ki.2011.406

[13] Wada T, Pippin JW, Terada Y, Shankland SJ (2005) The cyclin-dependent kinase inhibitor p21 is required for TGF-beta1-induced podocyte apoptosis. Kidney Int 68: 1618–1629. 16164639 10.1111/j.1523-1755.2005.00574.x

[14] Schiffer M, Bitzer M, Roberts IS, Kopp JB, ten Dijke P, et al (2001) Apoptosis in podocytes induced by TGF-beta and Smad7. J Clin Invest 108: 807–816. 11560950 10.1172/JCI12367 PMC200928

[15] Susztak K, Raff AC, Schiffer M, Böttinger EP (2006) Glucose-induced reactive oxygen species cause apoptosis of podocytes and podocte depletion at the onset of diabetic nephropathy. Diabetes 55: 225–233. 16380497

[16] Marshall CB, Pippin JW, Krofft RD, Shankland SJ (2006) Puromycin aminonucleoside induces oxidant-dependent DNA damage in podocytes in vitro and in vivo. Kidney Int 70: 1962–1970. 17035936 10.1038/sj.ki.5001965

[17] Bridgewater DJ, Ho J, Sauro V, Matsell DG (2005) Insulin-like growth factors inhibit podocyte apoptosis through the PI3 kinase pathway. Kidney In*t* 67: 1308–1314. 15780083 10.1111/j.1523-1755.2005.00208.x

[18] Xiao H, Shi W, Liu S, Wang W, Zhang B, et al (2009) 1,25-Dihydroxyvitamin D(3) prevents puromycin aminonucleoside-induced apoptosis of glomerular podocytes by activating the phosphatidylinositol 3-kinase/Akt-signaling pathway. Am J Nephrol 30: 34–43. 19202327 10.1159/000200769

[19] Kopperud R, Krakstad C, Selheim F, Døskeland SO (2003) cAMP effector mechanisms. Novel twists for an old signaling system. FEBS Lett 546: 121–126. 12829247 10.1016/s0014-5793(03)00563-5

[20] Zhu C, Huang S, Yuan Y, Ding G, Chen R, et al (2011) Mitochondrial dysfunction mediates aldosterone-induced podocyte damage: a therapeutic target of PPARγ. Am J Pathol 178: 2020–2031. 21514419 10.1016/j.ajpath.2011.01.029 PMC3081205

[21] Martinou JC, Green DR (2001) Breaking the mitochondrial barrier. Nat Rev Mol Cell Biol 2: 63–67. 11413467 10.1038/35048069

[22] Zamzami N, Kroemer G (2001) The mitochondrion in apoptosis: how Pandora's box opens. Nat Rev Mol Cell Biol 2: 67–71. 11413468 10.1038/35048073

[23] Youle RJ, van der Bliek AM (2012) Mitochondrial fission, fusion, and stress. Science 337: 1062–1065. 22936770 10.1126/science.1219855 PMC4762028

[24] Gu L, Dai Y, Xu Jin, Mallipattu S, Kaufman L, et al (2013) Deletion of podocytes STAT3 mitigates the entire spectrum of HIV-1-associated nephropathy. AIDS 27: 1091–1098. 23343908 10.1097/QAD.0b013e32835f1ea1 PMC3918880

[25] Ruster C, Franke S, Wenzel U, Schmidthaupt R, Fraune C, et al (2011) Podocytes of AT2 receptor knockout mice are protected from angiotensin II-mediated RAGE induction. Am J Nephrol 34: 309–317. 21846974 10.1159/000329321

[26] Huber TB, Gloy J, Heneger A, Schollmeyer P, Greger R, et al (1998) Catecholamines modulate podocyte function. J Am Soc Nephrol 9: 335–345. 9513895 10.1681/ASN.V93335

[27] Bek M, Fischer KG, Greiber S, Hupfer C, Mundel P, et al (1999) Dopamine deploarizes podocytes via a D1-like receptor. Nephrol Dial Transplant 14: 581–587. 10193803 10.1093/ndt/14.3.581

[28] He JC, Lu TC, Fleet M, Sunamoto M, Husain M, et al (2007) Retinoic acid inhibits HIV-1-induced podocyte proliferation through the cAMP pathway. J Am Soc Nephrol 18: 93–102. 17182884 10.1681/ASN.2006070727 PMC3197239

[29] Gao SY, Li CY, Shimokawa T, Matsuda S, Yaoita E, et al (2007) Rho-family small GTPase are involved in forskolin-induced cell-cell contact formation of renal glomerular podoctes in vitro. Cell Tissue Res 328: 391–400. 17265067 10.1007/s00441-006-0365-3

[30] Keravis T, Monneaux F, Yougbaré I, Gazi L, Bourguignon JJ, et al (2012) Disease progression in MRL/lpr lupus-prone mice is reduced by NCS 613, a specific cyclic nucleotide phosphodiesterase type 4 (PDE4) inhibitor. PLoS One 7: e28899. 22247763 10.1371/journal.pone.0028899 PMC3256138

[31] Endlich N, Endlich K (2002) cAMP pathway in podocytes. Microsc Res Tech 57: 228–231. 12012389 10.1002/jemt.10079

[32] Zhong Y, Wu Y, Liu R, Deng Y, Mallipattu SK, et al (2012) Roflumilast enhances the renal protective effects of retinoids in an HIV-1 transgenic mouse model of rapidly progressive renal failure. Kidney Int 81: 856–864. 22258322 10.1038/ki.2011.467 PMC3326224

[33] Saito Y, Okamura M, Nakajima S, Hayakawa K, Huang T, et al (2010) Suppression of nephrin expression by TNF-alpha via interfering with the cAMP-retinoic acid receptor pathway. Am J Physiol Renal Physiol 298: F1436–F1444. 20237236 10.1152/ajprenal.00512.2009

[34] Oba S, Hino M, Fujita T (2008) Adrenomedullin protects against oxidative stress-induced podocyte injury as an endogenous antioxidant. Nephrol Dial Transplant 23: 510–517. 17981883 10.1093/ndt/gfm600

[35] Gloerich M, Bos JL (2010) Epac: defining a new mechanism for cAMP action. Annu Rev Pharmacol Toxicol 50: 355–375. 20055708 10.1146/annurev.pharmtox.010909.105714

[36] Li Y, Konings IB, Zhao J, Price LS, de Heer E, et al (2008) Renal expression of exchange protein directly activated by cAMP (Epac) 1 and 2. Am J Physiol Renal Physiol 295: F525–F533. 18495799 10.1152/ajprenal.00448.2007

[37] Qin Y, Stokman G, Yan K, Ramaiahgari S, Verbeek F, et al (2012) cAMP signaling protects proximal tubular epithelial cells from cisplatin-induced apoptosis via activation of Epac. Br J Pharmacol 165: 1137–1150. 21745194 10.1111/j.1476-5381.2011.01594.x PMC3346244

[38] Stokman G, Qin Y, Genieser HG, Schwede F, de Heer E, et al (2011) Epac-Rap signaling reduces cellular stress and ischemia-induced kidney failure. J Am Soc Nephrol 22: 859–872. 21493776 10.1681/ASN.2010040423 PMC3083308

[39] Faour WH, Gomi K, Kennedy CR (2008) PGE(2) induces COX-2 expression in podocytes via the EP(4) receptor through a PKA-independent mechanism. Cell Signal 20: 2156–2164. 18762248 10.1016/j.cellsig.2008.08.007

[40] Shen B, Kwan HY, Ma X, Wong CO, Du J, et al (2011) cAMP activates TRPC6 channel s via the phosphatidylinositol 3-kinase (PI3K)-protein kinase B (PKB)-mitogen-activated protein kinase kinase (MEK)-ERK1/2 signaling pathway. J Biol Chem 286: 19439–19445. 21487005 10.1074/jbc.M110.210294 PMC3103323

[41] Youle RJ, Karbowski M (2005) Mitochondrial fission in apoptosis. Nature Reviews Molecular Cell Biolog*y* 6: 657–663. 16025099 10.1038/nrm1697

[42] CowellRM, BlakeKR, InoueT, RussellJW (2008) Regulation of PGC-1 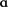 and PGC-1 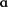 -responsive genes with forskolin-induced Schwann cell differentiation. Neurosci Lett 493: 269–274.10.1016/j.neulet.2008.04.104PMC258744318538475

[43] Cartoni R, Léger B, Hock MB, Praz M, Crettenand A, et al (2005) Mitofusins 1/2 and ERR alpha expression are increased in human skeletal muscle after physical exercise. J Physiol 567: 349–358. 15961417 10.1113/jphysiol.2005.092031 PMC1474174

